# An empowerment programme to support children in child headed households in resource-poor communities in Soshanguve, South Africa: phase 2 of an intervention study

**DOI:** 10.1186/1471-2334-14-S3-O29

**Published:** 2014-05-27

**Authors:** Julia E Ibebuike, Corrien van Belkum, Todd MM Maja

**Affiliations:** 1 Adelaide Tambo School of Nursing Science, Tshwane University of Technology, Pretoria, South Africa; 2 College of Nursing, King Saud bin Abdulazis University for Health Sciences, Riyadh, Saudi Arabia

## Background

South Africa has been noted to have the fastest growing rate of people living with HIV/AIDS. This has led to an alarming increase in the number of children orphaned by AIDS and the attendant care of the orphaned children by their siblings leading to the formation of child headed households (CHHs). The study was conducted in two phases. Phase 1 identified CHHs in a resource poor community in South Africa, their lived experiences, their needs and the resource poor communities’ knowledge and perceptions about these households. Phase 2 of the study aimed at developing, implementing and evaluating an empowerment programme to support the children in CHHs in the resource poor communities.

## Methods

The design for the study was a qualitative contextual intervention study and targeted children in CHHs who participated in Phase 1 of the study. Data was collected using in depth interviews, and data analysis was by open coding using Tesch’s approach.

## Results

The study findings revealed the following, (1) structure for developing an empowerment programme to support children in CHHs in resource poor communities, (2) framework for empowerment programme to support children in CHHs, (3) summary of the implementation of the framework for an empowerment programme to support children in CHHs, (4) evaluation of the implementation and outcome of the empowerment programme.

## Conclusion

An empowerment programme was developed, effectively implemented and the children’s needs supported.

